# LDAR is superior to other albumin-derived indices in predicting 28-day ICU mortality in critically ill patients with intracerebral hemorrhage: a two-cohort study

**DOI:** 10.3389/fnut.2026.1844200

**Published:** 2026-07-20

**Authors:** Ping Yang, Daizhou Peng, Zhigui Huang, Dengjian Luo, Li Dong, Zhiping Huang, Kaigu Li

**Affiliations:** 1 Department of Neurosurgery, The Affiliated Xingyi Hospital of Guizhou Medical University, Xingyi, China; 2 Department of Neurology, The Qianxinan People's Hospital, Xingyi, China; 3 Department of Pharmacy, The Affiliated Xingyi Hospital of Guizhou Medical University, Xingyi, China

**Keywords:** albumin-derived composite indices, correlation analysis, intracerebral hemorrhage, lactate dehydrogenase to albumin ratio, predictive performance comparison

## Abstract

**Background:**

Spontaneous intracerebral hemorrhage (ICH) carries a high risk of poor outcomes, making early identification of high-risk patients crucial for improving prognosis. Albumin-derived composite indices can reflect the systemic inflammatory, nutritional, and metabolic status, but their predictive performance in the ICH population lacks systematic comparison. This study aimed to evaluate the prognostic value of six albumin-derived composite indices for 28-day ICU mortality in critically ill patients with ICH.

**Methods:**

This study included 1,638 adult patients with first-time ICH admission from the MIMIC-IV database as the derivation cohort, and 493 patients with ICH from the People’s Hospital of Xingyi City as the external validation cohort. Clinical data within 24 h of admission were collected. Six indices were calculated: red blood cell distribution width to albumin ratio (RAR), creatinine to albumin ratio (CAR), anion gap to albumin ratio (AGAR), total bilirubin to albumin ratio (TAR), blood urea nitrogen to albumin ratio (UAR), and lactate dehydrogenase to albumin ratio (LDAR, log-transformed for analysis). Cox regression, restricted cubic splines (RCS), and Kaplan–Meier (KM) curves were used to analyze the association between each index and 28-day ICU mortality. Predictive performance was compared using receiver operating characteristic (ROC) curves. The incremental predictive value of log(LDAR) beyond traditional critical care scores was also assessed.

**Results:**

In the derivation cohort, 295 patients (18.0%) died within 28 days. After multivariable adjustment, log(LDAR) (HR = 1.68, 95%CI: 1.35–2.10), RAR (HR = 1.09, 95%CI: 1.01–1.19), and TAR (HR = 1.27, 95%CI: 1.16–1.40) were independent risk factors for 28-day mortality. RCS analysis revealed nonlinear associations with mortality risk for log(LDAR), TAR, and UAR (all P-non-linear < 0.05), whereas RAR showed a linear positive correlation (P-non-linear = 0.467). ROC curve analysis demonstrated that log(LDAR) had the highest predictive efficacy (AUC = 0.695), significantly outperforming the other indices (all DeLong test *p* < 0.05). Adding log(LDAR) to five traditional severity scores, including APACHE II and SOFA, significantly improved their predictive ability (AUC increase range: 0.016–0.039, all *p* < 0.05). Subgroup analyses confirmed the robustness of log(LDAR)’s predictive effect across different populations (all *p* for interaction > 0.05). These findings were validated in the external cohort.

**Conclusion:**

Among six albumin-derived composite indices, log(LDAR) offers superior predictive value for 28-day ICU mortality in critically ill ICH patients. It significantly enhances the risk stratification capability of traditional critical illness scores and may serve as a useful tool for early clinical screening.

## Introduction

Spontaneous intracerebral hemorrhage (ICH), a pathological condition resulting from the rupture of cerebral blood vessels with direct extravasation of blood into the brain parenchyma, is associated with high rates of mortality and disability, imposing a substantial burden on families and healthcare systems (1–3). Early identification, timely intervention, and accurate prognostic assessment are critical for reducing fatality rates, mitigating neurological damage, and managing complications (4, 5). Consequently, identifying effective biomarkers for predicting adverse outcomes in ICH has become a research priority. Although various clinical scoring systems have demonstrated correlations with ICH prognosis, their complexity and operational demands often limit widespread adoption in daily clinical practice (6–8). Thus, there is an urgent need for readily available biomarkers with robust predictive performance to aid clinicians in the early recognition of high-risk patients and to optimize treatment strategies.

Serum albumin, the most abundant protein in the human circulation, plays a pivotal role in maintaining colloid osmotic pressure and mediating anti-inflammatory and antioxidant processes. It is therefore considered a key clinical indicator for assessing nutritional status and systemic inflammatory burden (9, 10). Existing evidence links hypoalbuminemia to poor outcomes in various critical illnesses, including ICH (11–13). However, as a standalone predictor, serum albumin concentration is susceptible to confounding factors such as hepatic function, fluid balance, the acute-phase response, and clinical interventions (e.g., fluid resuscitation, nutritional support), which can compromise the stability and specificity of its prognostic value (14). To overcome the inherent limitations of single markers, recent research has increasingly focused on combining albumin with other pathophysiologically relevant and complementary laboratory parameters. Such composite indices may offer a more comprehensive picture of a patient’s inflammatory load, nutritional reserve, and metabolic stress, potentially enhancing clinical utility in ICH (15–18).

However, current evidence regarding albumin-derived composite indices for ICH prognosis predominantly stems from retrospective studies, and a lack of head-to-head comparisons among these indices leaves uncertainties regarding their differential predictive power and optimal clinical application. Given the significant resources required for prospective cohort studies, leveraging retrospective data to identify which integrated indices combine ease of use with stable predictive capacity is a necessary prerequisite. This approach can inform subsequent prospective study designs and optimize resource allocation. Therefore, this study aimed to systematically evaluate and compare the performance of several albumin-related inflammatory-nutritional composite indices in predicting 28-day ICU mortality among critically ill patients with ICH, with the goal of identifying the most promising candidate for clinical translation.

## Methods

### Data sources

This retrospective study utilized two cohorts. The derivation cohort was obtained from the publicly available Medical Information Mart for Intensive Care IV (MIMIC-IV) database (version 2.2) (19), which contains information on 196,527 adult patients admitted to the Beth Israel Deaconess Medical Center between 2008 and 2019. Database access was granted following an approved protocol. The Institutional Review Board of the Massachusetts Institute of Technology approved the use of the MIMIC-IV database for research and waived the requirement for informed consent due to the retrospective nature of the study and the use of de-identified data. The external validation cohort comprised patients with ICH treated at the Department of Neurosurgery, People’s Hospital of Xingyi City, enrolled between January 2015 and January 2025. This cohort was approved by the hospital’s ethics committee, which also waived the need for individual informed consent due to the retrospective study design.

### Study population

Data extraction from MIMIC-IV was performed using PostgreSQL (v13.7.2) and Navicat Premium (v16). Adult patients (age ≥ 18 years) admitted to the ICU with a diagnosis of ICH, identified by ICD-9 code 431 and ICD-10 codes I610–I619 and I62.9, were included. Exclusion criteria were: (1) death within 24 h of ICU admission, to avoid incomplete data or negative survival times; (2) multiple ICU admissions, retaining only the first ICH-related record to prevent data duplication; and (3) absence of key laboratory parameters required for calculating albumin-derived composite indices on the first ICU-time.

### Variable extraction and processing

Data extraction was performed using structured query language (SQL) via PostgreSQL (v13.7.2) and Navicat Premium (v16.0). Extracted variables encompassed six categories: (1) demographics; (2) comorbidities; (3) vital signs; (4) laboratory parameters; (5) disease severity scores; and (6) treatments received (detailed in Supplementary Tables S1, S2). For missing data, only variables with less than 30% missingness were imputed. Multiple imputation was performed using the ‘mice’ package (v3.16.0) in R, employing random forest models. The imputation model included all variables involved in the primary analyses: the continuous variables requiring imputation, fully observed key laboratory indicators, and the outcome variable.

### Exposures and clinical outcome

Based on previous literature (15–18, 20, 21), six albumin-derived composite indices were selected to comprehensively assess inflammatory activation, nutritional status, and metabolic stress: red blood cell distribution width to albumin ratio (RAR), creatinine to albumin ratio (CAR), anion gap to albumin ratio (AGAR), total bilirubin to albumin ratio (TAR), blood urea nitrogen to albumin ratio (UAR), and lactate dehydrogenase to albumin ratio (LDAR). These indices have been previously associated with poor ICH outcomes (15–18, 20, 21). To meet statistical assumptions and enhance clinical interpretability, LDAR was natural log-transformed and is presented as log(LDAR) throughout the analyses. The primary clinical endpoint was 28-day all-cause mortality following ICU admission.

### Association of albumin-derived indices with 28-day ICU mortality

To control for potential confounding factors, three sequentially adjusted models were constructed: Model 1 had no adjustments; Model 2 was adjusted for demographic variables (age, gender, race, body weight); Model 3 was further adjusted, based on previous literature (22–24), for a set of prespecified variables including major comorbidities (AKI, CKD, DM, IHD, COPD), vital signs (HR, NBPS, NBPD, RR), laboratory parameters (HCT, PLT, WBC, Ca, Glu, K, CO2CP, PH, PT, PTT, ALT, Mg), and treatments (SA, VP, CRRT, ventilation, GC, ABX). Additionally, Model 3 included components of five other albumin-derived indices (RDW, TB, CRE, AG, URE, LDH) to fully account for confounding. Importantly, to avoid multicollinearity with the index under investigation, when that index served as the exposure, the constituent variables of each specific albumin-derived index (e.g., LDH and albumin for LDAR; RDW and albumin for RAR; total bilirubin and albumin for TAR, etc.) were deliberately excluded from Model 3. Variance inflation factors (VIFs) were calculated for all remaining covariates, and no VIF exceeded 5, indicating no significant multicollinearity.

### Incremental value of log(LDAR)

Log(LDAR) was incorporated into five widely used critical illness scores within multivariable Cox models. Composite risk scores were calculated based on regression coefficients using the formula (*β*₁ × variable₁) + (β₂ × variable₂). Predictive performance for adverse outcomes was assessed using receiver operating characteristic (ROC) curves and the area under the curve (AUC). The DeLong test was used to compare model performance before and after adding log(LDAR).

### Subgroup and interaction analyses

Prespecified subgroup analyses and interaction tests were conducted to assess the robustness of the association between log(LDAR) and ICH outcomes. Subgroups were defined by key characteristics such as age, sex, race, and baseline comorbidities. Consistency of the log(LDAR) effect across subgroups was evaluated by testing the statistical significance of interaction terms (*p* for interaction), with a non-significant result (typically *p* > 0.05) supporting robustness and a significant result suggesting heterogeneity. All analyses followed a prespecified plan to control for bias from multiple comparisons.

### Statistical analysis

All continuous variables were first tested for normality using the Shapiro–Wilk test. The results showed that none of the continuous variables followed a normal distribution (all Shapiro–Wilk test *p*-values < 0.05). Therefore, continuous variables are presented as median (interquartile range, IQR), and comparisons between the two groups were performed using the Mann–Whitney U test. Categorical variables are presented as counts (percentages), and group comparisons were performed using the Pearson chi-square test or Fisher’s exact test (when expected frequencies were <5). All statistical analyses were conducted using R software (version 4.5.1), and a two-sided *p*-value < 0.05 was considered statistically significant.

## Results

### Baseline characteristics of the study population

Baseline characteristics stratified by 28-day ICU mortality status are shown in Table 1. Compared with the survival group, patients in the non-survival group were older (median 69.0 vs. 66.0 years, *p* = 0.005), had a different racial distribution (lower proportion of white race), and carried a higher comorbidity burden, with significantly higher incidences of AKI, CKD, DM, IHD, and COPD (all *p* < 0.05). Clinical severity scores (SOFA, APS III, SAPS II, OASIS, APACHE II, and Charlson Comorbidity Index) were significantly higher in the non-survival group than in the survival group (all *p* < 0.001). Regarding vital signs, the non-survival group had higher heart rate and respiratory rate, while systolic blood pressure, diastolic blood pressure, and mean arterial pressure were significantly lower (*p* ≤ 0.001). Laboratory findings showed that patients in the non-survival group had elevated levels of RDW, WBC, anion gap, potassium, glucose, magnesium, ALT, AST, total bilirubin, creatinine, blood urea nitrogen, LDH, INR, PT, and PTT, whereas levels of albumin, calcium, hemoglobin, red blood cell count, platelet count, hematocrit, bicarbonate, and pH were decreased (all *p* < 0.05). In terms of treatment, a higher proportion of patients in the non-survival group received mechanical ventilation, CRRT, vasoactive agents, and antibiotics (*p* ≤ 0.001). All six albumin-derived composite indices (RAR, CAR, AGAR, UAR, TAR, LDAR, and logLDAR) were significantly elevated in the non-survival group (all *p* < 0.001).

**Table 1:** Baseline characteristics of critically ill ICH patients in the internal derivation cohort, stratified by 28-day ICU survival status.

Variable	ALL	Survivor	No-survivor	*p* value
	*N* = 1,633	*N* = 1,338	*N* = 295	
RAR	4.25 [3.64;5.07]	4.12 [3.58;4.87]	4.83 [4.09;5.78]	<0.001
CAR	0.27 [0.21;0.38]	0.26 [0.20;0.35]	0.32 [0.23;0.55]	<0.001
AGAR	4.24 [3.57;5.17]	4.17 [3.50;5.00]	4.80 [3.93;5.90]	<0.001
UAR	5.00 [3.33;7.50]	4.67 [3.17;6.92]	6.67 [4.29;11.9]	<0.001
TAR	0.18 [0.11;0.29]	0.17 [0.11;0.26]	0.22 [0.15;0.47]	<0.001
logLDAR	4.38 [4.08;4.77]	4.33 [4.04;4.69]	4.65 [4.34;5.15]	<0.001
Age	67.0 [54.0;78.0]	66.0 [53.0;77.0]	69.0 [57.5;80.0]	0.005
Gender	978 (59.9%)	807 (60.3%)	171 (58.0%)	0.497
Race	887 (54.3%)	750 (56.1%)	137 (46.4%)	0.003
Weight	75.5 [64.3;90.0]	75.2 [64.2;89.7]	76.8 [65.0;92.9]	0.307
HTN	837 (51.3%)	697 (52.1%)	140 (47.5%)	0.168
AKI	447 (27.4%)	320 (23.9%)	127 (43.1%)	<0.001
CKD	216 (13.2%)	166 (12.4%)	50 (16.9%)	0.047
DM	381 (23.3%)	296 (22.1%)	85 (28.8%)	0.017
HLD	509 (31.2%)	412 (30.8%)	97 (32.9%)	0.528
IHD	360 (22.0%)	277 (20.7%)	83 (28.1%)	0.007
COPD	150 (9.19%)	113 (8.45%)	37 (12.5%)	0.036
SOFA	4.00 [2.00;6.00]	3.00 [2.00;5.00]	6.00 [3.00;9.00]	<0.001
APSIII	39.0 [29.0;52.0]	37.0 [28.0;48.0]	51.0 [39.0;67.0]	<0.001
SAPSII	34.0 [27.0;42.0]	32.0 [26.0;40.0]	40.0 [34.0;51.0]	<0.001
OASIS	32.0 [27.0;37.0]	31.0 [26.0;36.0]	36.0 [32.0;42.0]	<0.001
Charlson	5.00 [3.00;7.00]	5.00 [3.00;7.00]	6.00 [4.00;8.00]	<0.001
APACHEII	15.0 [11.0;20.0]	14.0 [11.0;19.0]	20.0 [15.0;25.0]	<0.001
HR	85.0 [73.0;98.0]	85.0 [72.0;96.0]	89.0 [74.0;103]	0.001
NBPS	130 [115;147]	131 [117;148]	129 [111;145]	0.011
NBPD	73.0 [62.0;85.0]	74.0 [63.0;85.0]	69.0 [57.5;82.0]	<0.001
NBPM	88.0 [78.0;100]	88.0 [78.0;101]	84.0 [72.0;98.0]	<0.001
RR	18.0 [16.0;22.0]	18.0 [15.0;22.0]	20.0 [17.0;24.0]	<0.001
SpO2	98.0 [96.0;100]	98.0 [96.0;100]	99.0 [96.0;100]	0.847
HCT	35.3 [30.8;39.1]	35.7 [31.5;39.3]	33.4 [27.8;38.0]	<0.001
Hb	11.7 [10.1;13.1]	11.8 [10.3;13.2]	10.9 [9.20;12.6]	<0.001
PLT	194 [142;255]	196 [148;256]	169 [110;248]	<0.001
RDW	13.9 [13.1;15.2]	13.8 [13.1;15.0]	14.6 [13.6;16.4]	<0.001
RBC	3.86 [3.31;4.35]	3.92 [3.39;4.37]	3.61 [3.01;4.18]	<0.001
WBC	10.7 [8.00;14.4]	10.4 [7.90;13.9]	12.9 [8.60;17.8]	<0.001
ALB	3.40 [3.00;3.80]	3.40 [3.00;3.80]	3.10 [2.70;3.60]	<0.001
AG	14.0 [12.0;17.0]	14.0 [12.0;17.0]	15.0 [12.0;18.0]	0.002
Ca	8.60 [8.10;9.00]	8.60 [8.10;9.10]	8.40 [7.90;8.90]	<0.001
Cl	104 [100;107]	104 [101;107]	104 [100;108]	0.972
Glu	131 [108;167]	128 [107;160]	148 [116;193]	<0.001
K	4.00 [3.60;4.40]	3.90 [3.60;4.30]	4.10 [3.70;4.50]	<0.001
Na	139 [137;142]	139 [137;142]	139 [136;142]	0.522
CO2CP	24.0 [22.0;27.0]	25.0 [22.0;27.0]	24.0 [20.0;27.0]	0.002
PCO2	39.0 [33.0;45.0]	38.5 [33.0;45.0]	39.0 [33.0;47.0]	0.487
PH	7.40 [7.34;7.45]	7.40 [7.35;7.45]	7.39 [7.31;7.44]	0.001
PO2	116 [73.0;189]	117 [74.0;193]	111 [71.5;181]	0.111
INR	1.20 [1.10;1.30]	1.20 [1.10;1.30]	1.30 [1.10;1.50]	<0.001
PT	13.0 [11.9;14.7]	12.8 [11.8;14.3]	14.0 [12.6;16.5]	<0.001
PTT	28.5 [25.8;31.9]	28.3 [25.8;31.3]	30.1 [26.4;35.3]	<0.001
ALT	24.0 [16.0;44.0]	24.0 [15.0;44.0]	26.0 [17.0;46.5]	0.008
AST	33.0 [21.0;60.0]	31.0 [21.0;54.0]	43.0 [26.0;87.5]	<0.001
TB	0.60 [0.40;1.00]	0.60 [0.40;0.90]	0.70 [0.50;1.30]	<0.001
CRE	0.90 [0.70;1.20]	0.90 [0.70;1.10]	1.00 [0.70;1.60]	<0.001
URE	16.0 [12.0;24.0]	16.0 [12.0;23.0]	21.0 [14.0;33.0]	<0.001
LDH	266 [209;365]	252 [202;341]	330 [264;470]	<0.001
Mg	1.90 [1.70;2.10]	1.90 [1.70;2.10]	1.90 [1.70;2.20]	0.028
Ventilation	1,298 (79.5%)	1,042 (77.9%)	256 (86.8%)	0.001
CRRT	73 (4.47%)	26 (1.94%)	47 (15.9%)	<0.001
SA	1,095 (67.1%)	841 (62.9%)	254 (86.1%)	<0.001
VP	695 (42.6%)	496 (37.1%)	199 (67.5%)	<0.001
GC	413 (25.3%)	336 (25.1%)	77 (26.1%)	0.780
AHT	1,125 (68.9%)	915 (68.4%)	210 (71.2%)	0.384
ABX	1,262 (77.3%)	1,009 (75.4%)	253 (85.8%)	<0.001

HTN, Hypertension; AKI, Acute Kidney Injury; CKD, Chronic Kidney Disease; DM, Diabetes Mellitus; HLD, Hyperlipidemia; HF, Heart Failure; SOFA, Sequential Organ Failure Assessment; APS III, Acute Physiology Score III; SAPS II, Simplified Acute Physiology Score II; OASIS, Oxford Acute Severity of Illness Score; APACHE II, Acute Physiology and Chronic Health Evaluation II; HR, Heart Rate; NBPS, Non-Invasive Blood Pressure (Systolic); NBPD, Non-Invasive Blood Pressure (Diastolic); NBPM, Non-Invasive Blood Pressure (Mean); RR, Respiratory Rate; SpO₂, Oxygen Saturation; HCT, Hematocrit; Hb, Hemoglobin; PLT, Platelet Count; RDW, Red Cell Distribution Width; RBC, Red Blood Cell Count; WBC, White Blood Cell Count; ALB, Albumin; AG, Anion Gap; Ca, Calcium; Cl, Chloride; Glu, Glucose; K, Potassium; Na, Sodium; PCO₂, Partial Pressure of Carbon Dioxide; pH, Potential of Hydrogen; PO₂, Partial Pressure of Oxygen; INR, International Normalized Ratio; PT, Prothrombin Time; PTT, Partial Thromboplastin Time; ALT, Alanine Aminotransferase; AST, Aspartate Aminotransferase; TB, Total Bilirubin; CRE, Creatinine; URE, Urea Nitrogen; LDH, Lactate Dehydrogenase; ALP, Alkaline Phosphatase; Mg, Magnesium; PHOS, Phosphorus; RAR, RDW to Albumin Ratio; AGAR, Anion Gap to Albumin Ratio; LAR, Lactate to Albumin Ratio; UAR, Urea Nitrogen to Albumin Ratio; TAR, Total Bilirubin to Albumin Ratio; LDAR, Lactate Dehydrogenase to Albumin Ratio; Log₂(LDAR), Log₂-transformed Lactate Dehydrogenase to Albumin Ratio; CRRT, Continuous Renal Replacement Therapy; SA, Sedative Administration; VP, Vasopressor; GC, Glucocorticoids; AHT, Antihypertensive Therapy.

### Association of albumin-derived indices with 28-day ICU mortality

Cox regression analyses revealed significant associations between several indices and mortality risk (Table 2). In the unadjusted Model 1, AGAR (HR = 1.13, 95% CI: 1.08–1.19), CAR (HR = 1.41, 95% CI: 1.25–1.58), log(LDAR) (HR = 1.83, 95% CI: 1.57–2.14), RAR (HR = 1.20, 95% CI: 1.13–1.28), TAR (HR = 1.32, 95% CI: 1.23–1.41), and UAR (HR = 1.03, 95% CI: 1.02–1.04) were all significantly associated with increased mortality risk (all *p* < 0.001). These associations remained significant in the partially adjusted Model 2. In the fully adjusted Model 3, log(LDAR) (HR = 1.68, 95% CI: 1.35–2.10, *p* < 0.001), RAR (HR = 1.09, 95% CI: 1.01–1.19, *p* = 0.037), and TAR (HR = 1.27, 95% CI: 1.16–1.40, *p* < 0.001) remained independently associated with ICU mortality, while AGAR, CAR, and UAR did not. These findings indicate that log(LDAR), RAR, and TAR possess independent prognostic value, with log(LDAR) demonstrating a particularly strong association after comprehensive adjustment.

**Table 2:** COX regression results of six albumin-derived indices with 28-day ICU mortality in critically ill patients with intracerebral hemorrhage.

	Model 1			Model 2			Model 3		
Characteristic	HR	95% CI	*p*-value	HR	95% CI	*p*-value	HR	95% CI	*p*-value
AGAR	1.13	1.08, 1.19	<0.001	1.14	1.09, 1.20	<0.001	1.03	0.96, 1.11	0.4
CAR	1.41	1.25, 1.58	<0.001	1.40	1.24, 1.59	<0.001	0.97	0.70, 1.33	0.8
Log(LDAR)	1.83	1.57, 2.14	<0.001	1.95	1.67, 2.28	<0.001	1.68	1.35, 2.10	<0.001
RAR	1.20	1.13, 1.28	<0.001	1.20	1.13, 1.28	<0.001	1.09	1.01, 1.19	0.037
TAR	1.32	1.23, 1.41	<0.001	1.38	1.29, 1.48	<0.001	1.27	1.16, 1.40	<0.001
UAR	1.03	1.02, 1.04	<0.001	1.03	1.02, 1.04	<0.001	1.02	1.00, 1.03	0.052

CI, Confidence Interval; HR, Hazard Ratio.

### Dose–response relationships between albumin-derived indices and 28-day ICU mortality

**Figure 1:**
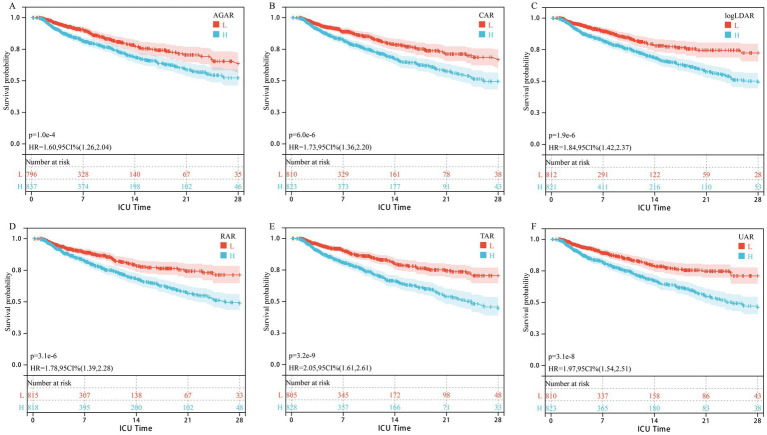
Kaplan–Meier survival curves for 28-day ICU mortality stratified by albumin-derived composite indices. Kaplan–Meier curves comparing cumulative survival probabilities over 28 days between patients stratified by the median value of each index: **(A)** RAR, **(B)** CAR, **(C)** AGAR, **(D)** log (LDAR), **(E)** TAR, and **(F)** UAR. Patients with index values above the median (high group) exhibited significantly lower survival rates compared to those with values below the median (low group) for all six indices. *p*-values were calculated using the log-rank test.

**Figure 2:**
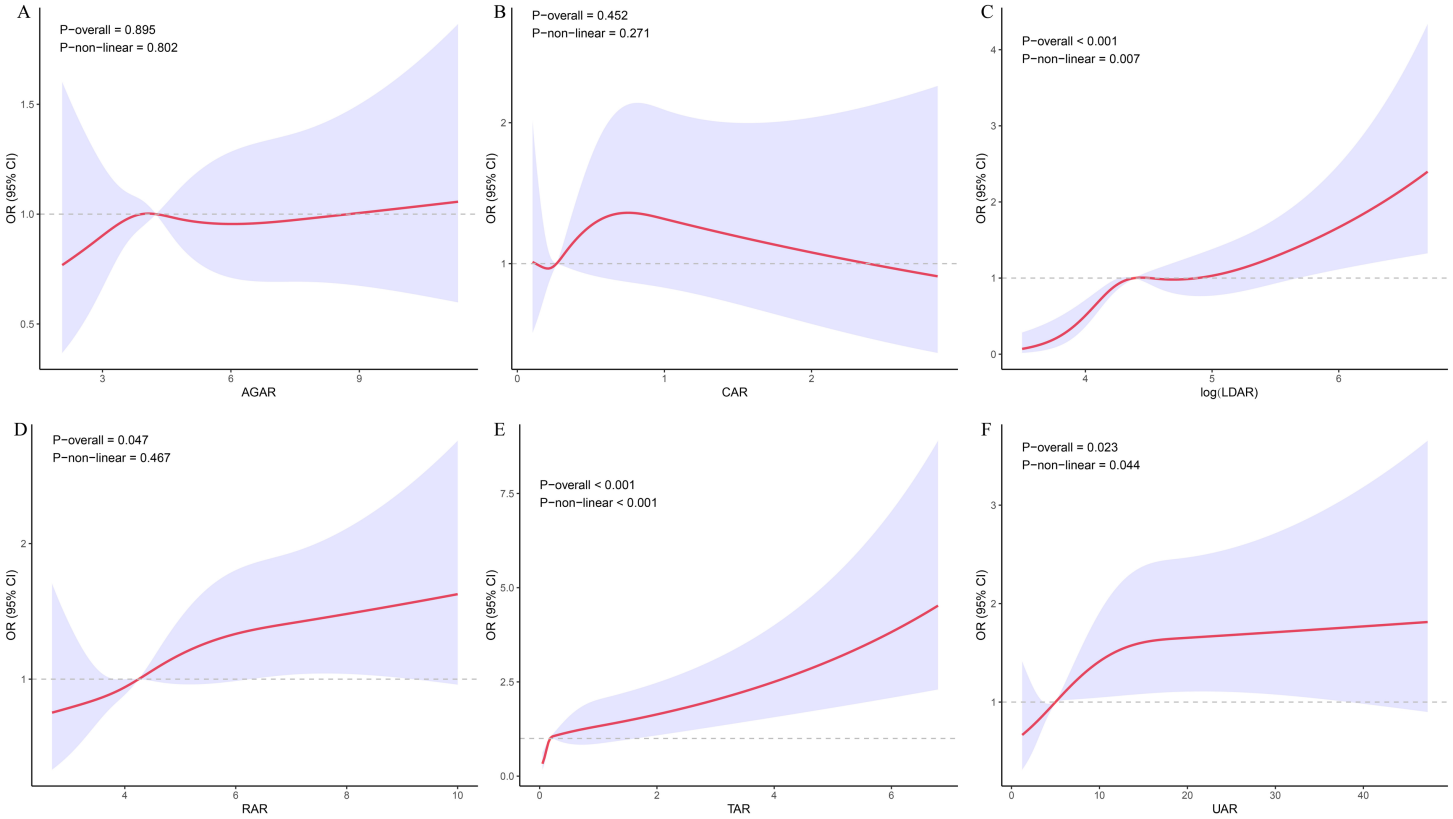
Dose–response relationships between albumin-derived composite indices and 28-day ICU mortality. Multivariable-adjusted restricted cubic spline (RCS) plots illustrating the hazard ratios (HRs) for 28-day ICU mortality associated with increasing levels of **(A)** AGAR, **(B)** CAR, **(C)** log(LDAR), **(D)** RAR, **(E)** TAR, and **(F)** UAR. Solid lines represent the adjusted HRs, and shaded areas indicate the 95% confidence intervals. Knots were placed at the 5th, 35th, 65th, and 95th percentiles. p-values for overall association and non-linearity are shown. RAR demonstrated a significant linear association, while log (LDAR), TAR, and UAR showed significant non-linear associations. AGAR and CAR were not significantly associated with mortality after adjustment.

Kaplan–Meier survival curves (Figure 1) demonstrated that patients with values above the median for each index (RAR, CAR, AGAR, log(LDAR), TAR, UAR) had significantly lower 28-day ICU survival rates compared to those below the median (all log-rank *p* < 0.001). Multivariable-adjusted restricted cubic spline (RCS) analyses (Figure 2) revealed distinct patterns of association. RAR showed a near-linear positive association with mortality risk (*p*-overall = 0.047, P-non-linear = 0.467; Figure 2D). In contrast, log(LDAR), TAR, and UAR exhibited significant non-linear associations (all P-non-linear < 0.05; Figures 2C,E,F), suggesting more complex dose–response relationships, potentially involving threshold effects or inflection points. AGAR and CAR showed no significant overall association after multivariable adjustment (*p*-overall > 0.05; Figures 2A,B). These results highlight the non-linear nature of some indices, which should be considered in risk stratification and model development.

### Comparative predictive performance of albumin-derived indices

**Figure 3:**
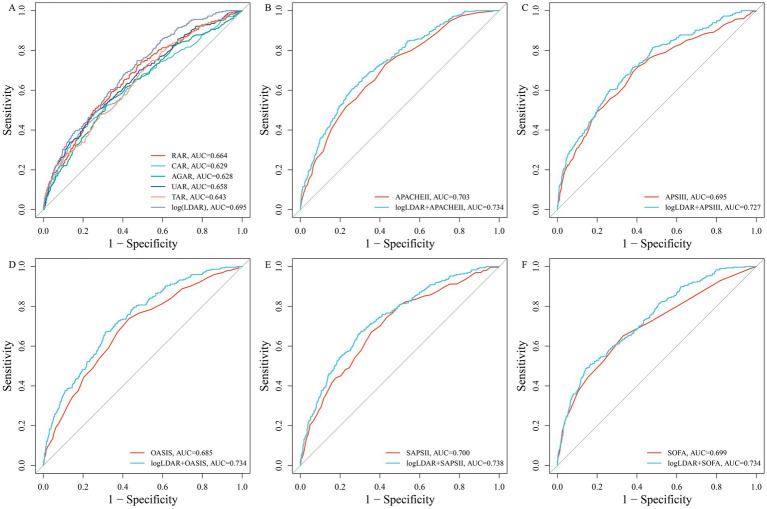
Predictive performance of albumin-derived indices and the incremental value of log (LDAR). Receiver operating characteristic (ROC) curves for predicting 28-day ICU mortality. **(A)** Comparison of the six albumin-derived composite indices. Log (LDAR) showed the highest area under the curve (AUC). **(B-F)** Incremental predictive value of log (LDAR) when added to five traditional critical illness severity scores: **(B)** APACHE II, **(C)** APS III, **(D)** SOFA, **(E)** SAPS II, and **(F)** OASIS. The addition of log (LDAR) significantly improved the AUC of each score (all DeLong test *p* < 0.05).

ROC curve analysis (Figure 3A) demonstrated significant differences in the discriminatory ability of the indices for ICU mortality (all DeLong test *p* < 0.05). Log(LDAR) exhibited the highest predictive efficacy (AUC = 0.695), followed by RAR (AUC = 0.664), UAR (AUC = 0.658), TAR (AUC = 0.643), CAR (AUC = 0.629), and AGAR (AUC = 0.628). These findings suggest that log(LDAR), RAR, and UAR offer valuable discriminatory power for prognostic assessment. Given its superior performance, log(LDAR) was selected for subsequent in-depth analyses.

### Subgroup and interaction analyses for log(LDAR)

**Figure 4:**
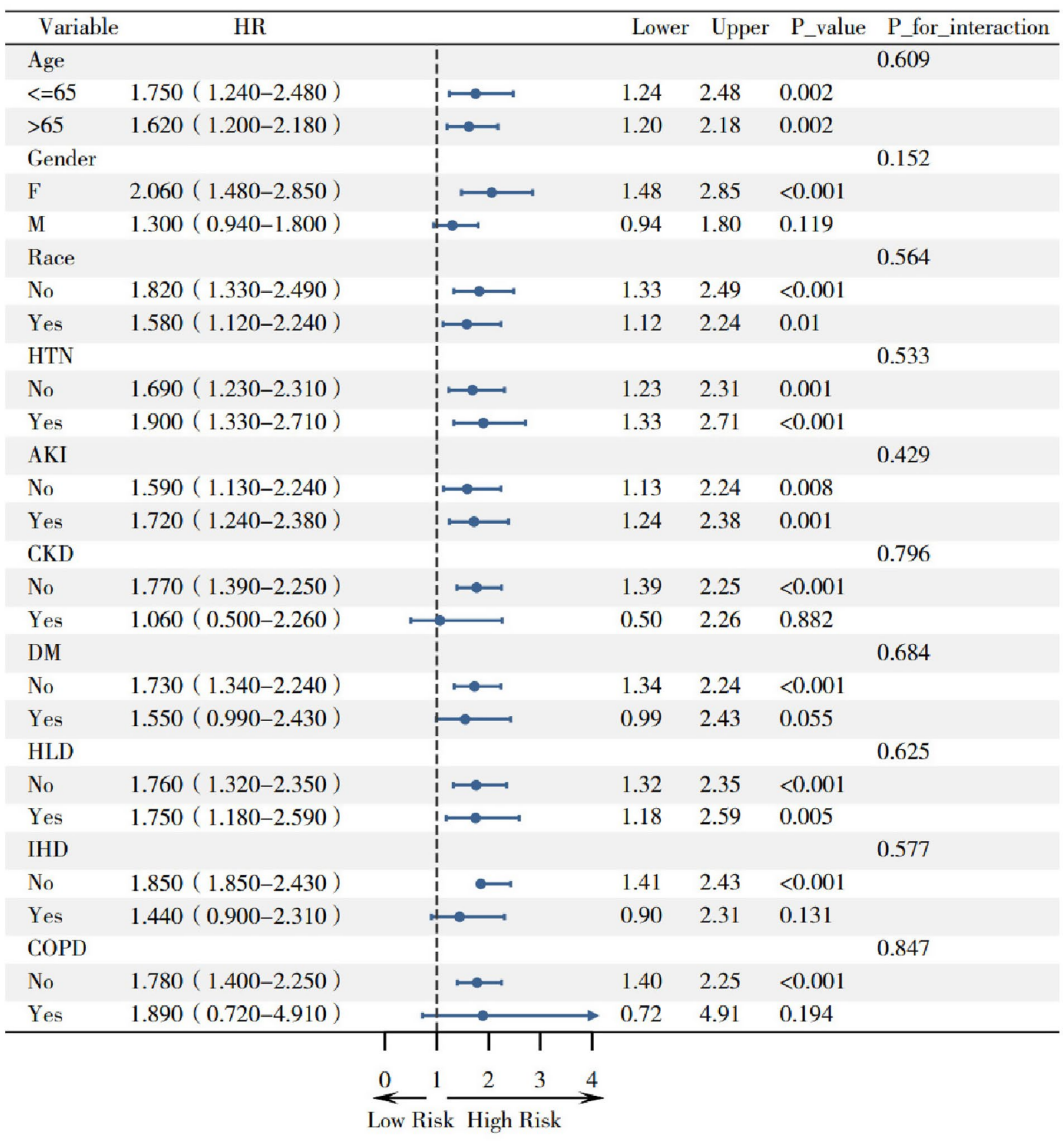
Subgroup analysis for the association between log (LDAR) and 28-day ICU mortality. Forest plot displaying the hazard ratios (HRs) for 28-day ICU mortality associated with log (LDAR) across various clinically relevant subgroups. HRs were calculated using multivariable-adjusted Cox regression models within each subgroup. The size of the blue squares is proportional to the number of patients in each subgroup, and horizontal lines represent the 95% confidence intervals. p-values for interaction are shown on the right, indicating no significant effect modification by any of the variables examined.

Subgroup and interaction analyses were conducted to assess the consistency of the log(LDAR)-mortality association across clinically relevant strata (Figure 4). Multivariable-adjusted models showed that log(LDAR) remained a significant predictor of mortality in most subgroups: age ≤ 65 years (HR = 1.75, *p* = 0.002), age > 65 years (HR = 1.62, p = 0.002), female sex (HR = 2.06, *p* < 0.001), various racial groups, and patients with or without hypertension, acute kidney injury, diabetes, hyperlipidemia, or ischemic heart disease (most *p* < 0.05). The association did not reach statistical significance in some subgroups, including males (HR = 1.30, *p* = 0.119), patients with chronic kidney disease (HR = 1.06, *p* = 0.882), diabetes (HR = 1.55, *p* = 0.055), or ischemic heart disease (HR = 1.44, *p* = 0.131), potentially due to smaller sample sizes or fewer events. Interaction tests revealed no significant effect modification by any of the variables examined (all *p* for interaction > 0.05), supporting the overall robustness of the log(LDAR)-mortality relationship across different patient populations.

### Incremental value of log(LDAR) beyond established severity scores

Incorporating log(LDAR) into five established critical illness severity scores consistently and significantly improved their predictive accuracy for ICU mortality (all DeLong test *p* < 0.05; Figures 3B–F). Specifically, the AUC for APACHE II increased from 0.703 to 0.734 (Figure 3B); for APS III, from 0.695 to 0.727 (Figure 3C); for SOFA, from 0.685 to 0.734 (Figure 3D); for SAPS II, from 0.700 to 0.738 (Figure 3E); and for OASIS, from 0.699 to 0.734 (Figure 3F). These findings demonstrate that adding log(LDAR) enhances the ability of existing clinical assessment tools to identify high-risk patients.

### External validation

**Figure 5:**
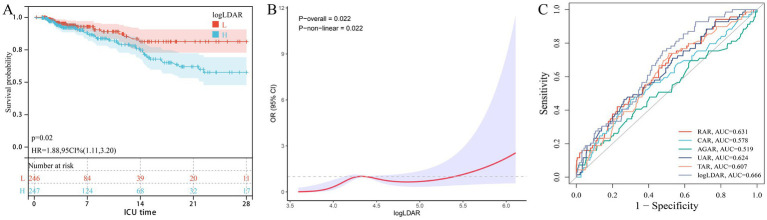
External validation of log(LDAR) for predicting 28-day ICU mortality. Validation of the prognostic value of log(LDAR) in an independent external cohort (*n* = 493). **(A)** Kaplan–Meier survival curves stratified by the median log(LDAR) value (log-rank *p* = 0.02). **(B)** Multivariable-adjusted restricted cubic spline plot showing a significant linear positive dose–response relationship between log(LDAR) and 28-day ICU mortality. **(C)** ROC curves comparing the predictive performance of log(LDAR) with other albumin-derived indices (RAR and TAR), confirming the superior discriminatory ability of log(LDAR) (AUC = 0.666) in the external cohort.

The external validation cohort comprised 493 critically ill patients with ICH (28-day mortality rate = 14.0%) (Supplementary Table S1). In a Cox regression model adjusted for all potential covariates, higher log(LDAR) remained significantly associated with increased 28-day mortality risk (HR = 2.10; 95% CI: 1.06–4.12, *p* = 0.032). Kaplan–Meier analysis confirmed significantly lower 28-day ICU survival in patients with log(LDAR) above the median (log-rank *p* = 0.02) (Figure 5A). RCS analysis in the external cohort revealed a significant linear positive dose–response relationship between log(LDAR) and short-term ICU mortality (Figure 5B). Replicating findings from the derivation cohort, LDAR demonstrated superior predictive ability (AUC = 0.666) compared to RAR (0.631), TAR (0.607), and the other albumin-derived indices in this external dataset (Figure 5C).

### Sensitivity analysis adjusting for ICH-specific variables in the external validation cohort

In the external cohort (*N* = 493), where data on hematoma volume, hemorrhage location, GCS score, antithrombotic therapy, and surgical intervention were fully available, we performed an additional multivariable Cox regression incorporating these five variables. After adjustment, log(LDAR) remained independently associated with 28-day ICU mortality (HR = 1.42, 95% CI: 1.08–1.86, *p* = 0.012). Hematoma volume (per 1 mL increase) was also a significant predictor (HR = 1.019, 95% CI: 1.007–1.031, *p* = 0.002), while GCS score (HR = 0.84, *p* < 0.001) and brainstem/cerebellar location (HR = 2.70, *p* = 0.008) showed expected associations. Compared with the main model (HR = 2.10), the effect direction of log (LDAR) remains consistent after adding these variables, which confirms the robustness of our research results.

### Clinical utility of log(LDAR)

To enhance clinical applicability, the optimal cut-off value for log(LDAR) predicting 28-day ICU mortality was determined in the derivation cohort (MIMIC-IV, n = 1,638) by maximizing the Youden index based on the ROC curve. The optimal cut-off was 4.351, yielding a sensitivity of 74.9%, specificity of 52.8%, and Youden index of 0.277. This indicates that at this threshold, log(LDAR) identifies approximately 75% of patients at risk of death while correctly ruling out about 53% of non-survivors. Applying the same cut-off (4.351) to the external validation cohort (n = 493) produced highly consistent results: sensitivity of 75.4%, specificity of 52.4%, and Youden index of 0.278. This consistency across cohorts supports the stability and generalizability of this cut-off and the reliability of log(LDAR) as a prognostic marker. However, the moderate specificity suggests that this marker alone is insufficient as a precise diagnostic or decision-making tool due to a relatively high false-positive rate.

## Discussion

Spontaneous ICH carries one of the highest mortality rates in neurocritical care, making early identification of high-risk patients and timely intervention crucial for improving outcomes (1–3). Although various clinical scoring systems exist, their complexity often limits routine clinical application (6–8). Consequently, identifying simple and reliable biomarkers holds significant clinical value. Serum albumin is a core indicator of nutritional and inflammatory status, but its use as a standalone marker is susceptible to confounding factors (14). Albumin-derived composite indices, which integrate information from different pathophysiological dimensions, have recently shown promise in critical care prognostication (16–18). However, a systematic comparison of these indices to determine the most effective predictor specifically for the ICH population has been lacking.

In this study, we systematically evaluated the predictive performance of six albumin-derived composite indices for 28-day ICU mortality in critically ill patients with ICH. Our results demonstrated that log(LDAR), RAR, and TAR were independent predictors of mortality after adjusting for multiple potential confounders. Notably, log(LDAR) exhibited the strongest discriminatory ability, with an area under the receiver operating characteristic curve (AUC = 0.695) significantly superior to the other indices (RAR: 0.664, UAR: 0.658, TAR: 0.643). This superior predictive performance of log(LDAR) was robustly validated in an independent external cohort (AUC = 0.666), where it continued to outperform the other five albumin-derived indices. Furthermore, integrating log(LDAR) into five traditional critical illness scoring systems, including APACHE II and SOFA, significantly enhanced their discriminatory power (all DeLong test *p* < 0.05). For instance, the AUC for APACHE II increased from 0.703 to 0.734, and for SOFA from 0.685 to 0.734. Subgroup analyses revealed that the predictive effect of log(LDAR) was generally consistent across diverse demographic and comorbidity strata. Although statistical significance was not reached in some subgroups (e.g., males, patients with chronic kidney disease, diabetes, or ischemic heart disease), potentially due to limited sample sizes, interaction tests revealed no significant effect modification (all *p* for interaction > 0.05). This suggests the prognostic value of log(LDAR) is broadly robust across different patient populations. Collectively, this evidence underscores the potential of log(LDAR) as a simple, effective, and novel biomarker for early risk stratification in critically ill patients with ICH.

The superior predictive performance of log(LDAR) compared to the other five albumin-derived indices in this study likely stems from its unique molecular composition, which simultaneously captures two critical pathophysiological dimensions following ICH: the severity of acute brain tissue damage and the depletion of systemic homeostatic reserve. Specifically, this index integrates information from LDH and albumin. LDH is a cytoplasmic enzyme widely distributed in tissues such as the brain, muscle, and liver. It is released into the peripheral blood following compromised cell membrane integrity, serving as a sensitive marker of cellular necrosis and tissue damage (25, 26). In the pathological process of ICH, hematoma formation and subsequent secondary injuries (e.g., excitotoxicity, oxidative stress, and inflammatory cascades) lead to extensive neuronal and glial cell necrosis, thereby releasing LDH. Thus, LDH levels directly reflect the extent of brain tissue necrosis and are closely associated with early hematoma expansion and poor outcomes (25–27). Furthermore, as a key enzyme in the lactate-pyruvate metabolic cycle, elevated LDH activity can promote reactive oxygen species (ROS) generation, exacerbating oxidative stress and consequently worsening neurological deficits post-ICH (28, 29). Recent research also indicates a role for LDH in maintaining vascular homeostasis; inhibiting LDH activity may exert neuroprotective effects by mitigating secondary brain injury, suggesting it is not merely a damage marker but also a potential therapeutic target (26, 30).

On the other hand, serum albumin, the primary plasma protein synthesized by the liver, plays a crucial role in maintaining colloid osmotic pressure, mitigating oxidative stress, and modulating inflammatory responses (9, 10). Its level is not only a key indicator of nutritional status but also a comprehensive reflection of systemic inflammatory burden and hepatic synthetic function (9, 10). In the pathophysiology of ICH, hypoalbuminemia and inflammation engage in a bidirectional vicious cycle: low albumin states are often accompanied by malnutrition and inflammatory activation, while persistent inflammation further suppresses albumin synthesis, exacerbating nutritional depletion and organ injury (9, 10, 31). Additionally, hypoalbuminemia can compromise antioxidant capacity, promote lipid peroxidation, and impair immune function, thereby aggravating secondary brain injury and systemic complications following ICH (32–39).

Therefore, by combining LDH and albumin, log(LDAR) effectively identifies a high-risk clinical phenotype characterized by “severe injury coupled with depleted reserve.” High LDH represents the acute, “fuel-on-the-fire” injurious process, while low albumin signifies the exhaustion of the body’s “firefighting capacity.” This dual imbalance synergistically drives the progression of malignant cerebral edema and the development of systemic complications, ultimately leading to a sharply increased mortality risk. In contrast, while indices like RAR (primarily reflecting red cell heterogeneity and anemia) or UAR (reflecting prerenal azotemia) possess predictive value, they do not directly capture the core injurious event of “cellular disintegration” in the same way as LDAR, which may fundamentally explain their comparatively lower predictive efficacy.

Furthermore, it is clinically relevant to discuss the optimal cut-off value for log(LDAR) identified in this study. By maximizing the Youden index in the derivation cohort (MIMIC-IV, *n* = 1,638), we determined the optimal threshold for predicting 28-day ICU mortality in critically ill ICH patients to be 4.351. This threshold demonstrated remarkable consistency between the derivation cohort (sensitivity 74.9%, specificity 52.8%) and the external validation cohort (75.4, 52.4%), confirming its robustness and generalizability. These data suggest that the approximate 75% sensitivity indicates a low risk of missed diagnosis, making it potentially useful as an early screening tool. However, the approximate 52% specificity implies a relatively high false-positive rate, precluding its use as a standalone decision-making tool. Thus, a log(LDAR) value > 4.351 should be considered a warning signal, prompting a more comprehensive clinical evaluation (e.g., incorporating GCS score, imaging findings, and traditional critical illness scores). Future research should focus on the dynamic trajectory of log(LDAR) and its combination with other markers to develop individualized predictive models.

Compared to previous studies (15–18), this investigation possesses several methodological strengths. First, to our knowledge, this is the first study to perform a head-to-head comparison of six albumin-derived indices specifically within a critically ill ICH population, rather than validating a single marker in isolation. Our dual-cohort analysis definitively establishes the superiority of log(LDAR) in predicting 28-day ICU mortality (AUC = 0.695, outperforming RAR, UAR, etc.), providing direct evidence to inform biomarker selection in clinical practice. Second, regarding analytical depth, we not only validated the independent prognostic value of log(LDAR) (HR = 1.68) and its incremental value over traditional critical illness scores (AUC increase 0.016–0.039) but also confirmed its general robustness across diverse populations via subgroup analyses (all interaction *p* > 0.05). Additionally, our use of restricted cubic spline analysis revealed a non-linear relationship between log(LDAR) and mortality risk, deepening the understanding of its clinical significance. Third, rigorous validation in an external real-world cohort demonstrated high consistency in the predictive performance (AUC = 0.666) and the optimal cut-off value (4.351) for log(LDAR), confirming its strong robustness and generalizability.

Despite these meaningful findings, several limitations warrant acknowledgment. First, the retrospective design, while effective for exploring associations, cannot entirely exclude residual confounding or establish causality; thus, prospective studies are needed to validate our conclusions. Second, all analyses were based on single laboratory measurements obtained within 24 h of admission, precluding dynamic monitoring of key indices during the ICU stay, although longitudinal changes might contain richer prognostic information. Third, while using ICD codes to identify ICH patients is standard in database research, the potential for misclassifying a small number of non-traumatic ICH cases cannot be entirely ruled out. However, given the rarity of such cases in a critically ill population and the highly consistent findings between internal and external cohorts, this potential bias likely has a limited impact. Fourth, although the MIMIC-IV derivation cohort lacked structured data on key ICH-specific prognostic factors (including hematoma volume, bleeding site, admission GCS score, anticoagulation therapy, and surgical intervention), we addressed this limitation in the external validation cohort. In the external cohort (*N* = 493), all these variables were fully available. Sensitivity analyses incorporating them into the fully adjusted model confirmed that log(LDAR) remained independently associated with 28-day ICU mortality (HR = 1.42, 95% CI: 1.08–1.86, *p* = 0.012). Although the effect size was moderately lower than that in the original model (HR = 2.10), the direction of effect remained consistent. Nonetheless, we acknowledge that the inability to adjust for these variables in the derivation cohort remains a limitation, and residual confounding cannot be completely ruled out. Future prospective studies with standardized collection of imaging and treatment data are warranted to further validate our findings.

## Conclusion

In this two-cohort retrospective analysis systematically comparing six albumin-derived composite indices for predicting 28-day ICU mortality in critically ill patients with ICH, log(LDAR) emerged as the most effective independent predictor. Log(LDAR) not only significantly enhanced the discriminatory ability of traditional critical illness scoring systems, including APACHE II and SOFA, but also demonstrated robust predictive performance across diverse demographic and comorbidity subgroups. Consistent internal and external validation confirmed the superior predictive efficacy of log(LDAR) over the other five albumin-derived indices. These findings suggest that log(LDAR) may serve as a simple and cost-effective biomarker to aid early identification of high-risk ICH patients and optimize risk assessment in clinical practice. However, its clinical utility and generalizability require further validation in future multicenter prospective studies.

## Data Availability

The original contributions presented in the study are included in the article/Supplementary material, further inquiries can be directed to the corresponding author/s.
