# Supplementary material for: LDAR is superior to other albumin-derived indices in predicting 28-day ICU mortality in critically ill patients with intracerebral hemorrhage: a two-cohort study
Source: Front Nutr. 2026 Jul 20;13:1844200. doi: 10.3389/fnut.2026.1844200 (PMC13429447; doi:10.3389/fnut.2026.1844200)
Supplement: Supplementary file 1 [file Table_1.docx]

**Table S1. Baseline characteristics of critically ill ICH patients in the external validation cohort, stratified by 28-day ICU survival status**

| **Variable** | **ALL** | **Survivor** | **No-survivor** | **P value** |
| --- | --- | --- | --- | --- |
|  | ***N=493*** | ***N=424*** | ***N=69*** |  |
| RAR | 4.15 [3.59;4.82] | 4.09 [3.54;4.72] | 4.41 [3.95;5.30] | <0.001 |
| CAR | 0.26 [0.20;0.36] | 0.26 [0.20;0.35] | 0.29 [0.21;0.45] | 0.038 |
| AGAR | 4.17 [3.48;5.00] | 4.17 [3.47;5.00] | 4.29 [3.51;5.24] | 0.621 |
| UAR | 5.00 [3.33;7.19] | 4.85 [3.18;6.93] | 5.94 [4.21;10.0] | 0.001 |
| TAR | 0.17 [0.11;0.26] | 0.17 [0.11;0.25] | 0.21 [0.14;0.30] | 0.004 |
| LDAR | 79.0 [58.8;118] | 75.4 [56.5;115] | 95.5 [77.6;157] | <0.001 |
| logLDAR | 4.37 [4.07;4.77] | 4.32 [4.04;4.75] | 4.56 [4.35;5.06] | <0.001 |
| Age | 68.0 [53.0;78.0] | 68.0 [53.0;78.0] | 72.0 [60.0;83.0] | 0.016 |
| Gender: | 289 (58.6%) | 254 (59.9%) | 35 (50.7%) | 0.192 |
| BMI | 28.0 [24.4;30.6] | 28.5 [24.7;30.6] | 27.7 [23.9;30.6] | 0.422 |
| HTN: | 253 (51.3%) | 214 (50.5%) | 39 (56.5%) | 0.422 |
| AKI: | 128 (26.0%) | 98 (23.1%) | 30 (43.5%) | 0.001 |
| CKD: | 66 (13.4%) | 54 (12.7%) | 12 (17.4%) | 0.388 |
| DM: | 111 (22.5%) | 95 (22.4%) | 16 (23.2%) | 1.000 |
| HLD: | 146 (29.6%) | 123 (29.0%) | 23 (33.3%) | 0.557 |
| COPD: | 49 (9.94%) | 37 (8.73%) | 12 (17.4%) | 0.044 |
| SOFA | 4.00 [2.00;6.00] | 3.00 [2.00;5.25] | 5.00 [3.00;7.00] | <0.001 |
| APSIII | 39.0 [28.0;51.0] | 37.5 [27.0;49.0] | 48.0 [33.0;59.0] | <0.001 |
| SAPSII | 34.0 [27.0;43.0] | 33.0 [26.0;41.0] | 39.0 [33.0;47.0] | <0.001 |
| OASIS | 32.0 [27.0;37.0] | 31.0 [27.0;37.0] | 35.0 [33.0;40.0] | <0.001 |
| APACHEII | 15.0 [11.0;20.0] | 14.0 [11.0;19.0] | 18.0 [15.0;24.0] | <0.001 |
| HR | 84.0 [72.0;97.0] | 84.0 [72.0;96.0] | 84.0 [73.0;103] | 0.385 |
| NBPS | 130 [115;147] | 130 [115;147] | 132 [112;142] | 0.770 |
| NBPD | 72.0 [62.0;85.0] | 72.0 [62.8;85.0] | 69.0 [57.0;81.0] | 0.125 |
| NBPM | 87.0 [77.0;99.0] | 87.5 [78.0;99.2] | 83.0 [74.0;99.0] | 0.197 |
| RR | 18.0 [15.0;22.0] | 18.0 [15.0;22.0] | 18.0 [16.0;22.0] | 0.527 |
| SpO2 | 98.0 [96.0;100] | 98.0 [96.0;100] | 99.0 [94.0;100] | 0.421 |
| HCT | 35.6 [32.0;39.3] | 35.7 [32.2;39.3] | 33.9 [28.4;39.1] | 0.098 |
| Hb | 11.8 [10.4;13.1] | 11.9 [10.6;13.1] | 11.0 [9.40;12.8] | 0.026 |
| PLT | 193 [143;253] | 196 [143;256] | 177 [134;223] | 0.165 |
| RDW | 13.8 [13.1;15.0] | 13.7 [13.1;14.9] | 14.4 [13.4;15.9] | 0.005 |
| RBC | 3.93 [3.46;4.33] | 3.96 [3.49;4.34] | 3.67 [3.04;4.30] | 0.055 |
| WBC | 10.8 [7.80;14.0] | 10.5 [7.80;13.8] | 12.6 [8.30;16.1] | 0.031 |
| ALB | 3.40 [3.00;3.80] | 3.40 [3.10;3.80] | 3.30 [2.90;3.70] | 0.005 |
| AG | 14.0 [12.0;17.0] | 14.0 [12.0;17.0] | 13.0 [11.0;16.0] | 0.043 |
| Ca | 8.60 [8.10;9.00] | 8.60 [8.20;9.10] | 8.60 [8.00;8.90] | 0.109 |
| Cl | 104 [101;108] | 104 [101;107] | 105 [102;108] | 0.128 |
| Glu | 130 [108;162] | 126 [107;154] | 153 [118;196] | <0.001 |
| K | 3.90 [3.60;4.40] | 3.90 [3.60;4.40] | 4.10 [3.60;4.50] | 0.199 |
| Na | 140 [137;142] | 140 [137;142] | 139 [137;142] | 0.827 |
| CO2CP | 25.0 [22.0;27.0] | 25.0 [22.0;27.0] | 24.0 [22.0;27.0] | 0.288 |
| PCO2 | 38.0 [33.0;45.0] | 38.0 [33.0;44.2] | 39.0 [32.0;47.0] | 0.701 |
| PH | 7.40 [7.34;7.45] | 7.40 [7.34;7.45] | 7.40 [7.32;7.44] | 0.195 |
| PO2 | 112 [73.0;184] | 113 [73.0;186] | 110 [80.0;178] | 0.811 |
| INR | 1.20 [1.10;1.30] | 1.20 [1.10;1.30] | 1.30 [1.10;1.40] | 0.001 |
| PT | 12.9 [11.8;14.6] | 12.9 [11.8;14.4] | 13.9 [12.5;15.4] | 0.002 |
| PTT | 28.6 [25.8;31.6] | 28.6 [25.8;31.5] | 29.5 [25.9;34.6] | 0.159 |
| ALT | 25.0 [16.0;48.0] | 24.0 [16.0;47.0] | 32.0 [21.0;57.0] | 0.049 |
| AST | 34.0 [21.0;61.0] | 32.5 [21.0;59.0] | 43.0 [30.0;89.0] | 0.003 |
| TB | 0.60 [0.40;0.90] | 0.60 [0.40;0.83] | 0.70 [0.40;1.00] | 0.079 |
| CRE | 0.90 [0.70;1.20] | 0.90 [0.70;1.10] | 0.90 [0.70;1.30] | 0.389 |
| URE | 17.0 [12.0;23.0] | 16.0 [12.0;23.0] | 18.0 [14.0;27.0] | 0.010 |
| LDH | 269 [206;373] | 258 [201;365] | 304 [264;413] | <0.001 |
| Mg | 1.90 [1.70;2.10] | 1.90 [1.70;2.10] | 1.90 [1.80;2.10] | 0.658 |
| Ventilation: | 405 (82.2%) | 344 (81.1%) | 61 (88.4%) | 0.196 |
| CRRT: | 16 (3.25%) | 10 (2.36%) | 6 (8.70%) | 0.015 |
| SA: | 325 (65.9%) | 268 (63.2%) | 57 (82.6%) | 0.003 |
| VP: | 191 (38.7%) | 151 (35.6%) | 40 (58.0%) | 0.001 |
| ABX: | 374 (75.9%) | 316 (74.5%) | 58 (84.1%) | 0.118 |

Abbreviations: HTN: Hypertension; AKI: Acute Kidney Injury; CKD: Chronic Kidney Disease; DM: Diabetes Mellitus; HLD: Hyperlipidemia; HF: Heart Failure; SOFA: Sequential Organ Failure Assessment; APS III: Acute Physiology Score III; SAPS II: Simplified Acute Physiology Score II; OASIS: Oxford Acute Severity of Illness Score; APACHE II: Acute Physiology and Chronic Health Evaluation II; HR: Heart Rate; NBPS: Non-Invasive Blood Pressure (Systolic); NBPD: Non-Invasive Blood Pressure (Diastolic); NBPM: Non-Invasive Blood Pressure (Mean); RR: Respiratory Rate; SpO₂: Oxygen Saturation; HCT: Hematocrit; Hb: Hemoglobin; PLT: Platelet Count; RDW: Red Cell Distribution Width; RBC: Red Blood Cell Count; WBC: White Blood Cell Count; ALB: Albumin; AG: Anion Gap; Ca: Calcium; Cl: Chloride; Glu: Glucose; K: Potassium; Na: Sodium; Lac: Lactate; PCO₂: Partial Pressure of Carbon Dioxide; pH: Potential of Hydrogen; PO₂: Partial Pressure of Oxygen; INR: International Normalized Ratio; PT: Prothrombin Time; PTT: Partial Thromboplastin Time; ALT: Alanine Aminotransferase; AST: Aspartate Aminotransferase; TB: Total Bilirubin; CRE: Creatinine; URE: Urea Nitrogen; LDH: Lactate Dehydrogenase; ALP: Alkaline Phosphatase; Mg: Magnesium; PHOS: Phosphorus; RAR: RDW to Albumin Ratio; AGAR: Anion Gap to Albumin Ratio; LAR: Lactate to Albumin Ratio; UAR: Urea Nitrogen to Albumin Ratio; TAR: Total Bilirubin to Albumin Ratio; LDAR: Lactate Dehydrogenase to Albumin Ratio; Log₂(LDAR): Log₂-transformed Lactate Dehydrogenase to Albumin Ratio; CRRT: Continuous Renal Replacement Therapy; SA: Sedative Administration; VP: Vasopressor; GC: Glucocorticoids; AHT: Antihypertensive Therapy.
