# Supplementary material for: LDAR is superior to other albumin-derived indices in predicting 28-day ICU mortality in critically ill patients with intracerebral hemorrhage: a two-cohort study
Source: Front Nutr. 2026 Jul 20;13:1844200. doi: 10.3389/fnut.2026.1844200 (PMC13429447; doi:10.3389/fnut.2026.1844200)
Supplement: Supplementary file 2 [file Table_2.docx]

**Table S2. Distribution of ICH‑specific prognostic variables in the external validation cohort**

| **Variable** | **All (N=493)** | **Survivors (N=424)** | **Non‑survivors (N=69)** | **P value** |
| --- | --- | --- | --- | --- |
| **Hematoma volume (mL), median** | 22.0 [12.0, 38.0] | 21.0 [11.0, 34.0] | 45.0 [28.0, 65.0] | <0.001 |
| **Hemorrhage location, n (%)** |  |  |  | <0.001 |
| **Basal ganglia** | 260 (52.7) | 238 (56.1) | 22 (31.9) |  |
| **Lobar** | 158 (32.0) | 140 (33.0) | 18 (26.1) |  |
| **Thalamus** | 48 (9.7) | 35 (8.3) | 13 (18.8) |  |
| **Brainstem/cerebellum** | 27 (5.5) | 11 (2.6) | 16 (23.2) |  |
| **Admission GCS score, median** | 13 [9, 15] | 14 [11, 15] | 6 [4, 9] | <0.001 |
| **Pre‑admission antithrombotic therapy, n (%)** | 40 (8.1) | 35 (8.3) | 5 (7.2) | 0.78 |
| **Surgical intervention (evacuation/EVD), n (%)** | 94 (19.1) | 88 (20.8) | 6 (8.7) | 0.019 |
